# Do distribution volumes and clearances relate to tissue volumes and blood flows? A computer simulation

**DOI:** 10.1186/1471-2253-6-7

**Published:** 2006-06-13

**Authors:** Jan FA Hendrickx, Hendrikus JM Lemmens, Steven L Shafer

**Affiliations:** 1 Department of Anesthesia, Stanford University School of Medicine, Stanford, California, USA; 2 Department of Biopharmaceutical Science, University of California at San Francisco, San Francisco, California, USA

## Abstract

**Background:**

Kinetics of inhaled agents are often described by physiological models. However, many pharmacokinetic concepts, such as context-sensitive half-times, have been developed for drugs described by classical compartmental models. We derived classical compartmental models that describe the course of the alveolar concentrations (F_A_) generated by the physiological uptake and distribution models used by the Gas Man^® ^program, and describe how distribution volumes and clearances relate to tissue volumes and blood flows.

**Methods:**

Gas Man^® ^was used to generate F_A _vs. time curves during the wash-in and wash-out period of 115 min each with a high fresh gas flow (8 L.min^-1^), a constant alveolar minute ventilation (4 L.min^-1^), and a constant inspired concentration (F_I_) of halothane (0.75%), isoflurane (1.15%), sevoflurane (2%), or desflurane (6%). With each of these F_I_, simulations were ran for a 70 kg patient with 5 different cardiac outputs (CO) (2, 3, 5, 8 and 10 L.min^-1^) and for 5 patients with different weights (40, 55, 70, 85, and 100 kg) but the same CO (5 L.min^-1^). Two and three compartmental models were fitted to F_A _of the individual 9 runs using NONMEM. After testing for parsimony, goodness of fit was evaluated using median prediction error (MDPE) and median absolute prediction error (MDAPE). The model was tested prospectively for a virtual 62 kg patient with a cardiac output of 4.5 L.min^-1 ^for three different durations (wash-in and wash-out period of 10, 60, and 180 min each) with an F_I _of 1.5% halothane, 1.5% isoflurane, sevoflurane 4%, or desflurane 12%.

**Results:**

A three-compartment model fitted the data best (MDPE = 0% and MDAPE ≤ 0.074%) and performed equally well when tested prospectively (MDPE ≤ 0.51% and MDAPE ≤ 1.51%). The relationship between CO and body weight and the distribution volumes and clearances is complex.

**Conclusion:**

The kinetics of anesthetic gases can be adequately described e by a mammilary compartmental model. Therefore, concepts that are traditionally thought of as being applicable to the kinetics of intravenous agents can be equally well applied to anesthetic gases. Distribution volumes and clearances cannot be equated to tissue volumes and blood flows respectively.

## Background

Like intravenous anesthetics, inhaled anesthetic agents can and are being safely administered without the knowledge of any pharmacokinetic model. In addition, the MAC and MAC awake concept link the end-expired concentrations (F_A_) with the clinically two most relevant endpoints, immobility and unconsciousness. Kinetic models can nevertheless be useful. Models are used to explain differences in wash-in and wash-out characteristics of different agents that may be relevant to clinical practice. Uptake models have also been used to try to predict the relationship between fresh gas flow, and delivered, inspired (F_I_), and end-expired concentrations of anesthetic agents in the commonly used circle breathing system [1]. Once a kinetic model has been developed, it can be linked to clinically relevant endpoints (immobility and unconsciousness) by incorporating an effect site compartment.

Physiological models are appealing because they can be understood in terms of anatomy, physiology, and physics. The kinetics of inhaled agents are commonly described using physiological modeling because of the availability of values for tissue partition coefficients, tissue volumes and organ blood flows. Yet many assumptions underlie these models, such as the absence of an arterial-end-expired gradient or the assumption that uptake and distribution in an organ is uniform and instantaneous as implicitly assumed by flow limited uptake and the use of a single value for tissue partition coefficients [2]. In addition, it is mathematically impossible to accurately fit all these parameters to "uptake" as it is clinically available in the operating room: the product of inspired concentration (F_I_) and ventilation minus the product of the end-expired concentration (F_A_) and ventilation. A parsimonious empirical polyexponential model ("classical compartmental modeling") similar to that used to describe the course of the concentration over time of intravenous agents may be better suited to analyze the partial pressure (or infusion rate) of potent inhaled anesthetics over time [3-5]. The first goal of this manuscript is to examine whether the kinetics of four different anesthetic agents generated by a physiological uptake model (used by the Gas Man^® ^program, Med Man Simulations, Inc., Chestnut Hill, MA) can be equally well described by a mammilary compartmental model (derived using non-linear mixed effect modeling or NONMEM) [6]. If so, concepts applied to intravenous agents, like the context-sensitive half time, would also be applicable to inhaled agents. The derived compartmental model will be prospectively tested against the physiological model used by the Gas Man^® ^program. More detail about both models is provided in the Appendix. The second goal of this manuscript is to address the controversial issue whether or to what an extent distribution volumes and clearances relate to tissue volumes and blood flows. We therefore examined whether distribution volumes and clearances match the tissue volumes and blood flows from the physiological model used to generate the data from which the parameters of the compartmental model were derived.

## Methods

### Part I: Deriving the kinetic parameters of the compartmental model

Gas Man^® ^was used to generate F_A _for 4 different agents (halothane, isoflurane, sevoflurane, and desflurane) during a wash-in and washout period of 115 min each with a high fresh gas flow (8 L.min-1) and a constant alveolar minute ventilation (4 L.min^-1^, regardless of patient weight). The default settings were used for the circuit and tissue composition. The inspired concentrations of halothane, isoflurane, sevoflurane, and desflurane were maintained constant during the wash-in period at 0.75; 1.15; 2; and 6% respectively. With each of these inspired concentrations, simulations were run for a 70 kg patient with 5 different cardiac outputs (2, 3, 5, 8 and 10 L.min^-1^) and for 5 patients with different weights (40, 55, 70, 85, and 100 kg) but the same cardiac output (5 L.min^-1^). This yielded 9 unique simulations for each agent (note that each group includes a 70 kg patient with a cardiac output of 5 L.min^-1^, which is why there were not 10 unique simulations). Two and three compartmental models were fitted to F_A _of the individual 9 simulations using NONMEM (the original data and the NONMEM control code are available in the Web supplement). The total administered dose was entered in the NONMEM data file as the product of the inspired concentration and duration. A naïve pooled data fit was done to obtain starting values for the structural parameters (distribution volumes and clearances) for each of the four agents. The effect of weight and cardiac output on each of the structural parameters (covariate analysis) was assessed using the minimum objective function of NONMEM, -2 × Log Likelihood (-2LL), to decide whether to accept or omit a covariate. The results were then tested for parsimony by sequentially deleting each of the covariate parameters using the NONMEM objective function as the criteria to accept or delete any covariate parameter. Goodness of fit was evaluated using median prediction error (MDPE) and median absolute prediction error (MDAPE) as a measure for bias and accuracy respectively [7]. MDPE and MDAPE were calculated as (F_A_Gas Man - F_A_3compartmental model)/F_A_3compartmental model) and |(F_A_Gas Man - F_A_3compartmental model)/F_A_3compartmental model| respectively, and are expressed in %.

### Part II: Prospective testing of the compartmental model

Using the previously derived parameters with their covariates, the compartmental model was tested prospectively by having it generate F_A _for a patient with parameters different from those from which the model was derived. The F_A _output for a 62 kg patient with a cardiac output of 4.5 L.min^-1 ^for three different durations (wash-in and wash-out period of 10, 60, and 180 min each) with an F_I _of 1.5% halothane, 1.5% isoflurane, sevoflurane 4%, or desflurane 12% was calculated by converting the volume and clearance parameters, including any covariate effects, into the coefficients and exponents defining the three exponential functions that together describe the F_A _course. Conversions were done with the Excel spreadsheet CONVERT.XLS [8]. The same patient parameter set was entered into Gas Man^® ^and the respective end-expired concentrations were obtained. The predictive performance of the compartmental model was tested using MDPE and MDAPE as described above. MDPE and MDAPE less than 20% have been considered acceptable. All data and control files are available upon simple request to the authors (JHX).

## Results

### Part I

**Figure 1:**
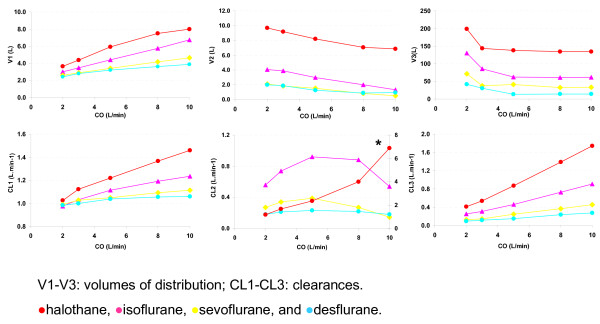
**Structural parameters for each of the four agents in a 70 kg patient with a cardiac output ranging from 2 to 10 L.min-1 for a 115 min wash-in and wash-out period**. * : denotes use of a second Y-axis for halothane (for clarity).

**Figure 2:**
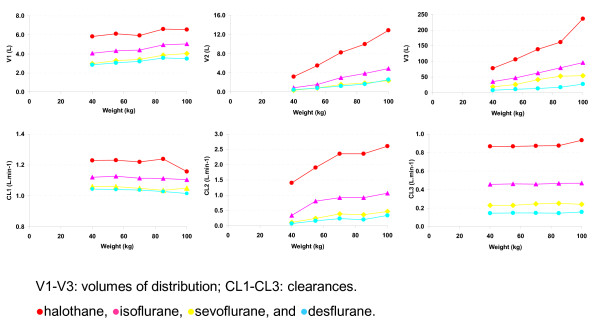
Structural parameters for each of the four agents in a patient with a cardiac output of 5 L.min-1 and weight ranging from 40 to 100 kg for a 115 min wash-in and wash-out period.

A three compartment model best described the F_A _course. The distribution volumes and clearances for each of the initial 9 simulation runs are presented in figure 1 and 2. The pharmacokinetic parameters for the three compartmental model with cardiac output and weight as covariates (tested for parsimony) are presented in (Table 1 and 2). As shown in table 2, the models described the data almost perfectly (MDPE = 0% and MDAPE ≤ 0.074%), indicating that a 3 compartment mammilary model is sufficient to capture the pharmacokinetic behavior of the anesthetic gases in a high flow open circuit.

**Table 1:** The pharmacokinetic parameters for the three compartmental model with cardiac output (CO, L.min-1) and weight (WT, kg) as covariates (tested for parsimony).

**Halothane**
V1 = 5.65 + 0.020(WT-70) + 0.68(CO-5)
V2 = 8.35 + 0.15(WT-70) - 0.49(CO-5)
V3 = 134.09 + 1.91(WT-70)
CL1 = 1.23 + 0.050(CO-5)
CL2 = 2.57 + 0.010(WT-70) + 0.47(CO-5)
CL3 = 0.87 + 0.17(CO-5)

**Isoflurane**

V1 = 4.21 + 0.020(WT-70) + 0.39(CO-5)
V2 = 3.16 + 0.065(WT-70) - 0.31(CO-5)
V3 = 61.40 + 0.87(WT-70)
CL1 = 1.12 + 0.024(CO-5)
CL2 = 1.05 + 0.0037(WT-70) + 0.17(CO-5)
CL3 = 0.46 + 0.092(CO-5)

**Sevoflurane**

V1 = 3.33 + 0.018(WT-70) + 0.21(CO-5)
V2 = 1.51 + 0.033(WT-70) - 0.18(CO-5)
V3 = 33.69 + 0.47(WT-70)
CL1 = 1.05 + 0.011(CO-5)
CL2 = 0.37 + 0.0034(WT-70) + 0.053(CO-5)
CL3 = 0.23 + 0.046(CO-5)

**Desflurane**

V1 = 2.94 + 0.017(WT-70) + 0.14(CO-5)
V2 = 1.56 + 0.030(WT-70) - 0.14(CO-5)
V3 = 14.08 + 0.19(WT-70)
CL1 = 1.03 + 0.0066(CO-5)
CL2 = 0.25 + 0.0022(WT-70) + 0.036(CO-5)
CL3 = 0.14 + 0.029(CO-5)

V = distribution volume (L), CL = clearance (L.min-1), with numbers 1, 2, and 3 referring to the first, second, and third compartment.

**Table 2:** Goodness of fit for the predictions by three compartment model predictions during model derivation and prospective testing.

	Model derivation		Prospective testing	
	MDPE	MDAPE	MDPE	MDAPE

Halothane	0.00	0.18	0.31	0.38
Isoflurane	0.00	0.29	0.38	0.52
Sevoflurane	0.00	0.63	0.27	0.73
Desflurane	0.00	0.74	0.51	1.51

MDPE = median prediction error; MDAPE = median absolute prediction error. Values are expressed in %.

### Part II

**Figure 3:**
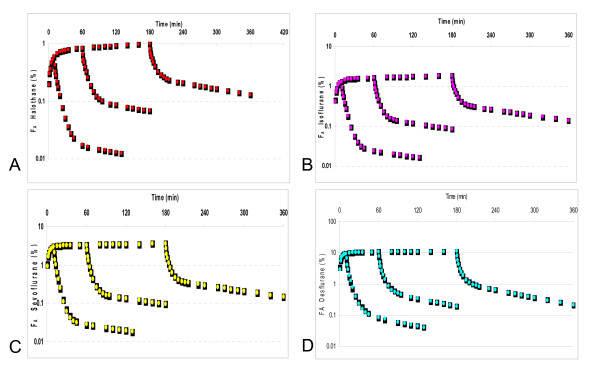
Prospective testing of the model: the end-expired concentrations (FA) predicted by the compartmental model (full line) and the corresponding values simulated by Gas Man^® ^(open circles) for halothane (A), isoflurane (B), sevoflurane (C), and desflurane (D).

F_A _of each of the four agents generated by the compartmental model and the corresponding values from Gas Man^® ^are illustrated in figure 3. Goodness of fit was excellent (MDPE ≤ 0.51% and MDAPE ≤ 1.51%) (Table 2).

## Discussion

The time course of F_A _of a potent inhaled anesthetic generated by a physiological uptake model (Gas Man^®^) can be equally well described using a classical three compartmental model derived from data generated by the very same physiological model (Gas Man^®^). While the relationship between the distribution volumes and clearances and organ capacities and tissue blood flows is complex, it is nevertheless intuitively tempting to try to explain e.g. the associations between a decrease in cardiac output and the changes in the volumes of distribution in terms of alterations in tissue blood flow of distributions. Yasuda and colleagues used NONMEM derived classical compartmental parameters from end-expired concentrations and related the parameters of the exponential curves (exponents and time constants) to known physiological processes and anatomical compartments [9]. These parameters were subsequently used to help delineate several physiological compartments: the first, second, third, fourth, and fifth compartment were interpreted as representing lungs, vessel rich group, muscle group, intertissue diffusion, and the fat group. Hull, however, acclaims that while it is often suggested that some particular tissue or organ (such as the brain) be 'in' one compartment or another, such suggestions are ill-founded because parameters of the fit to the uptake data contain no information that might support such assumptions [10,11]. Similarly, Wissing argues that a precise allocation of several hypothetical peripheral compartments to anatomical defined tissues is hardly feasible [2,3].

If it is true that distribution volumes and clearances can be directly interpreted in terms of tissue volumes and tissue blood flows, a change in weight or cardiac output that is known to cause proportional changes in tissue volumes or tissue blood flow in the (physiological) model of Gas Man^® ^should also cause proportional changes in the distribution volumes and clearances of the empirical compartmental (NONMEM) model. In our current simulations, an increase in cardiac output and weight does indeed increase clearance and distribution volume (Figures 1 and 2). However, this type of relationship is not consistent. An increase in cardiac output is associated with a decrease in the clearance of V2 (the second compartment) with sevoflurane and isoflurane. In addition, the non-linear (almost hyperbolic) relationship between V3 (the third compartment) and cardiac output for all four gases cannot be interpreted in terms of an underlying physiological model or process. Therefore, the parameters of the underlying physiological model (organ capacities and blood flows) and those of an empirical model (distribution volumes and clearances) therefore cannot be interpreted in terms of one another.

We have shown that the kinetics of anesthetic gases can be described by a mammilary model. Therefore, concepts that are traditionally thought of as being applicable to the kinetics of intravenous agents can be equally well applied to anesthetic gases. One example of one of these concepts are context sensitive half-times, which interestingly had been described for inhaled agents before the term context sensitive half-time was introduced but were labeled "coasting times" by Lowe and Ernst [12,13].

This study does not address when or whether to use physiological modeling or classical compartmental modeling. Either modeling approach adequately describes and predicts the course of F_A_, but their parameters cannot be interpreted in terms of one another. A three compartment model can be a good fit of a concentration that is effectively the sum of a multi-organ (physiological) system, where each organ displays first order kinetics [14]. This study does not address the validity of the GasMan^® ^model either. Even though GasMan^® ^can be used to gain additional insights into the kinetic of inhaled agents, clinical validation of the GasMan^® ^model is lacking.

## Conclusion

We describe how non-linear mixed-effect modeling can be used to derive a three compartmental empirical model to describe the kinetic behavior of potent inhaled anesthetic generated by a physiological model (Gas Man^®^). Therefore, concepts that are traditionally thought of as being applicable to the kinetics of intravenous agents can be equally well applied to anesthetic gases. The relationship between the distribution volumes and clearances of the empirical model and tissue capacities and blood flow of the physiological model are complex, however. Our study reinforces the fact that the compartmental models (even with co-variate models) are an empirical description of a data set, and that the parameters have virtually no physiological interpretation.

## Appendix. Graphical display and formulas of the empirical three-compartmental model (NONMEM) and the physiological model of Gas Man^®^

### The empirical three-compartmental model (NONMEM)

**Figure 4:**
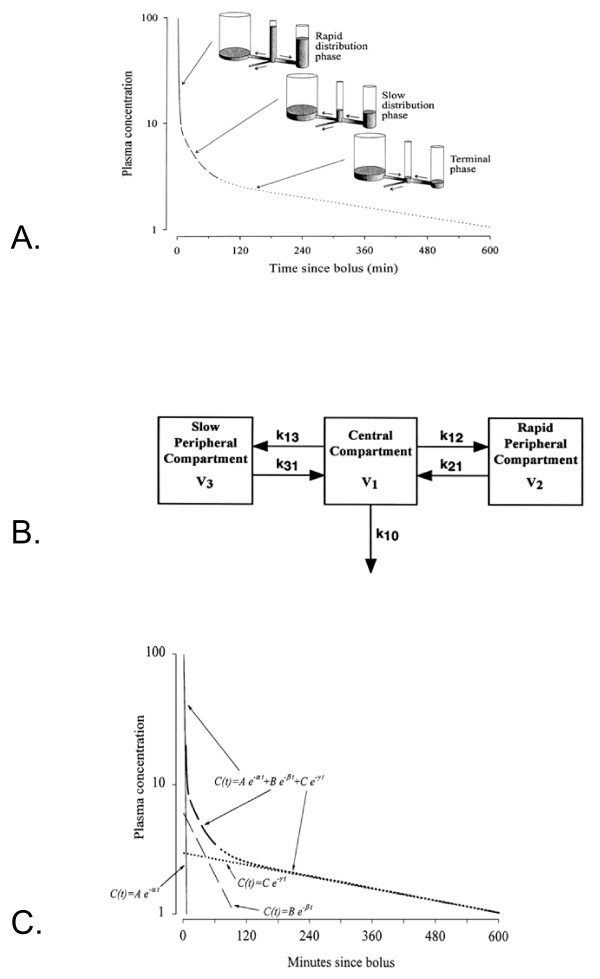
A. The three compartment empirical model: the volume-clearance scheme. B. The three compartment empirical model: the rate constant-scaling factor scheme. C. The three compartment empirical model: the mathematical three exponential scheme.

With a three compartmental model, the time course of the drug's concentration can be described in three mathematically equivalent ways: (a) three volumes and three clearances; (b) five rate constants and a scaling factor; or (c) a tri-exponential equation. The volume-clearance scheme is useful to visualize how the drug moves throughout the body (figure 4A). In the rate constant-scaling factor scheme (figure 4B) each "micro-rate constant" k_ij _defines the rate of drug transfer from one compartment i to another compartment j. V_1 _is the scaling factor; V_2 _and V_3 _are not independent parameters here. The mathematical form of the three-compartment exponential equation is most commonly used (figure 4C). A, B, and C are called "coefficients"; α, β, and γ (occasionally called λ_1_, λ_2_, and λ_3_) are "exponents" or "hybrid rate constants". While the volume-clearance and the rate constant-scaling factor scheme parameters are mathematically easily converted into one another [8], the coefficients and exponentials of the tri-exponential fit are related to the volumes and clearances and to the rate constants and scaling factor in a slightly more complex manner.

By using a sum of least squares method (the minimum objective function or MOF), NONMEM determines whether a one, two or three compartmental model best describes the concentration course. Depending on the type of control file used, NONMEM analysis will yield either the exponents and coefficients or distribution volumes and clearances. The best fitting model is then optimized by mathematically linking patient covariates (e.g. weight, height, cardiac output) to those parameters, again using the MOF to decide whether or not a covariate significantly improves the fit.

### The physiological model of Gas Man^®^

**Figure 5:**
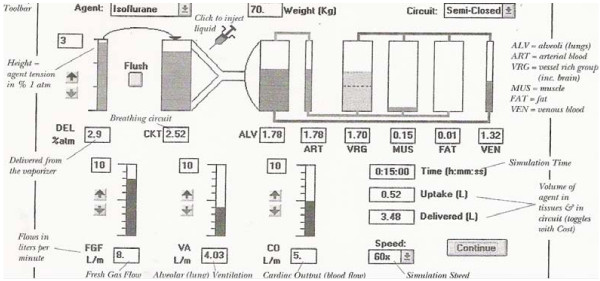
The Gas Man^® ^graphical interface.

The building blocks for the physiological model of Gas Man^® ^are also labeled "compartments". In Gas Man^®^, the patient is modeled as four compartments: the lung, vessel rich group, muscle and fat; the circuit is a fifth. Each patient compartment refers to a group of tissues for which uptake can be described by a differential equation if tissue volume, tissue flow, and tissue solubility are known. The model assumes that anesthetic uptake by an organ is a perfusion-limited, exponential process. Organ vapor capacity depends on the size of the organ and the solubility of the agent in that particular organ. The rate at which the partial pressure in the organ increases, and eventually saturates (=equals the partial pressure in the arterial blood), depends both on the organ's capacity (the more can be stored at the same partial pressure, the longer this will take), and on organ blood flow (the higher blood flow, the faster it will equilibrate with the arterial partial pressure, and therefore the faster it saturates). Based on both tissue storage capacity and blood flow, organs and tissue groups are grouped into the VRG, MG, and FG group (vessel rich group, muscle group, and fat group, respectively). The Gas Man^® ^simulation uses Euler's method of solution for the simultaneous differential equations, with linear coefficients that govern the five-compartment system. The model utilizes standard values for organ volumes, anesthetic solubilities, and regional blood flows. An example of the actual display is depicted in figure 5.

## Competing interests

The author(s) declare that they have no competing interests.

## Authors' contributions

All authors have contributed equally to all parts of the manuscript.

## Pre-publication history

The pre-publication history for this paper can be accessed here:


